# Supplementary material for: Development of Effective PEDV Vaccine Candidates Based on Viral Culture and Protease Activity
Source: Vaccines (Basel). 2023 Apr 30;11(5):923. doi: 10.3390/vaccines11050923 (PMC10222049; doi:10.3390/vaccines11050923)
Supplement: Supplementary file 1 [file vaccines-11-00923-s001.zip › vaccines-2363027-supplementary.pdf]

<u>**Supplementary Materials**</u>

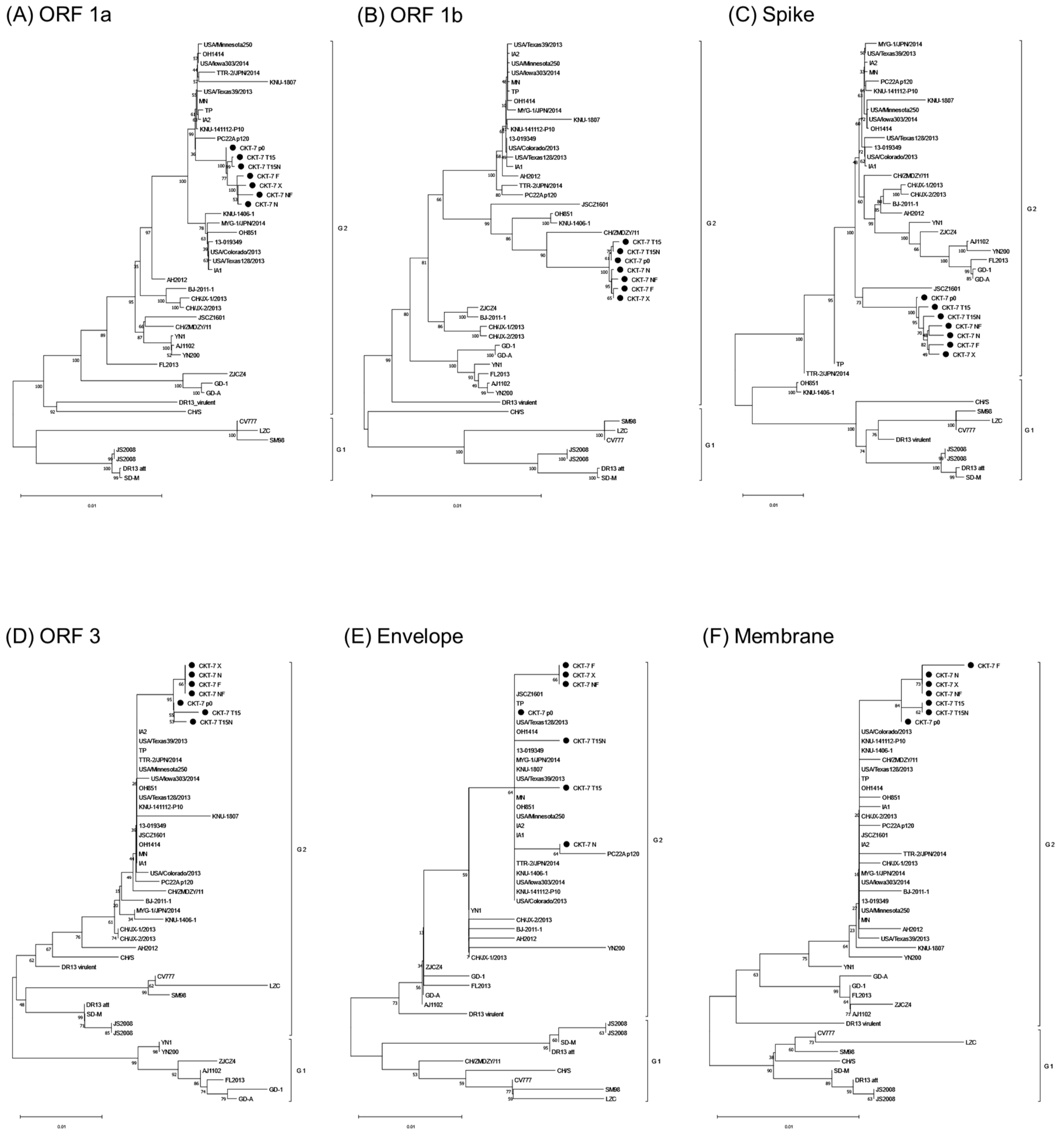

(G) Nucleocapsid

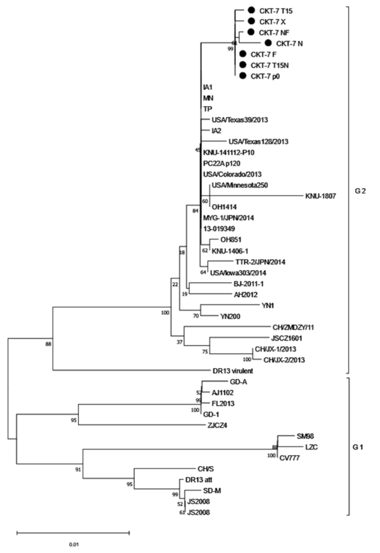

Figure S1. Analysis of phylogenetic relationship based on the nucleotide sequence of the ORF1a, ORF1b, spike (S), ORF3, envelope (E), membrane (M), and N genes. Phylogenetic tree of (A) the ORF1a gene; (B) the ORF1b gene; (C) the S gene; (D) the ORF3 gene; (E) the E gene; (F) the M gene; (G) the N gene. The trees were determined using the neighbor-joining method of MEGA X software. The bootstrap analysis was generated using 1000 replicates.

Table S1. Analysis of genomic alteration depend on strains

Gene	Nucleotide	Amino-acid position	PEDV strains						
			Parental	T15	T15N	N	NF	F	X
ORF 1a/b	4-6	2	GCT (A)	GCT (A)	GCT (A)	GCT (A)	--- (-)	GCT (A)	GCT (A)
	7-9	3	AGC (S)	AGC (S)	AGC (S)	AGC (S)	--- (-)	AGC (S)	AGC (S)
	10-12	4	AAC (N)	AAC (N)	AAC (N)	AAC (N)	--- (-)	AAC (N)	AAC (N)
	13-15	5	CAT (H)	CAT (H)	CAT (H)	CAT (H)	--- (-)	CAT (H)	CAT (H)
	16-18	6	GTT (V)	GTT (V)	GTT (V)	GTT (V)	TTA (L)	GTT (V)	GTT (V)
	19-21	7	ACA (T)	ACA (T)	ACA (T)	ACA (T)	CAT (H)	ACA (T)	ACA (T)
	23	8	TTG (L)	TTG (L)	TTG (L)	TTG (L)	TGG (W)	TTG (L)	TTG (L)
	25-26	9	GCT (A)	GCT (A)	GCT (A)	GCT (A)	CTT (L)	GCT (A)	GCT (A)
	31-33	11	GCC (A)	GCC (A)	GCC (A)	GCC (A)	AAA (K)	GCC (A)	GCC (A)
	34-36	12	AAT (N)	AAT (N)	AAT (N)	AAT (N)	CGA (R)	AAT (N)	AAT (N)
	37-39	13	GAT (D)	GAT (D)	GAT (D)	GAT (D)	CCG (P)	GAT (D)	GAT (D)
	40	14	GCA (A)	GCA (A)	GCA (A)	GCA (A)	TCA (S)	GCA (A)	GCA (A)
	43	15	GAA (E)	GAA (E)	GAA (E)	GAA (E)	CAA (Q)	GAA (E)	GAA (E)
	518	173	ACA (T)	ACA (T)	ACA (T)	ACA (T)	ACA (T)	ATA (I)	ATA (I)
	536	179	ACT (T)	ATT (I)	ACT (T)	ACT (T)	ACT (T)	ACT (T)	ACT (T)
	833	278	TGC (C)	TGC (C)	TGC (C)	TTC (F)	TTC (F)	TGC (C)	TTC (F)
	1601	534	CGC (R)	CGC (R)	CTC (L)	CGC (R)	CGC (R)	CGC (R)	CGC (R)
	2254	752	GTT (V)	GTT (V)	TTT (F)	GTT (V)	GTT (V)	GTT (V)	GTT (V)
	2815	939	AAG (K)	AAG (K)	AAG (K)	AAG (K)	GAG (E)	AAG (K)	AAG (K)
	2858	953	GTT (V)	GTT (V)	GTT (V)	GCT (A)	GTT (V)	GTT (V)	GTT (V)
	2941	981	CCC (P)	TCC (S)	TCC (S)	TCC (S)	TCC (S)	TCC (S)	TCC (S)
	3329	1110	GCA (A)	GCA (A)	GCA (A)	GCA (A)	GCA (A)	GCA (A)	GTA (V)
	4106	1369	GGT (G)	GGT (G)	GGT (G)	GAT (D)	GAT (D)	GAT (D)	GAT (D)
	4156	1386	GAG (E)	GAG (E)	GAG (E)	GAG (E)	GAG (E)	CAG (Q)	GAG (E)
	4369-4371	1457	TTT (F)	TTT (F)	TTT (F)	--- (-)	TTT (F)	TTT (F)	TTT (F)
	4592	1531	ACT (T)	AAT (N)	AAT (N)	ACT (T)	ACT (T)	ACT (T)	ACT (T)
	4691	1564	TCT (S)	TCT (S)	TCT (S)	TTT (F)	TTT (F)	TTT (F)	TTT (F)

	4715	1572	CCT (P)	CCT (P)	CCT (P)	CCT (P)	CCT (P)	CCT (P)	CTT (L)
	4718	1573	GAC (D)	GAC (D)	GAC (D)	GCC (A)	GCC (A)	GCC (A)	GCC (A)
	5062	1688	AAT (N)	AAT (N)	AAT (N)	AAT (N)	GAT (D)	AAT (N)	AAT (N)
	7306	2436	CTT (L)	CTT (L)	CTT (L)	CTT (L)	CTT (L)	CTT (L)	TTT (F)
	8129	2710	ATC (I)	ATC (I)	ATC (I)	ACC (T)	ACC (T)	ACC (T)	ACC (T)
	8712	2904	GAG (E)	GAG (E)	GAG (E)	GAT (D)	GAG (E)	GAG (E)	GAG (E)
	8841	2947	AAT (N)	AAT (N)	AAT (N)	AAT (N)	AAT (N)	AAG (K)	AAT (N)
	8924	2975	GAT (D)	GAT (D)	GAT (D)	GAT (D)	GAT (D)	GAT (D)	GGT (G)
	9264	3088	AAT (N)	AAT (N)	AAT (N)	AAG (K)	AAG (K)	AAG (K)	AAG (K)
	9973	3325	GTT (V)	TTT (F)	TTT (F)	GTT (V)	GTT (V)	GTT (V)	GTT (V)
	10634	3545	GCT (A)	GTT (V)	GTT (V)	GCT (A)	GCT (A)	GCT (A)	GCT (A)
	12380	4127	AAC (N)	AAC (N)	AAC (N)	AAC (N)	ATC (I)	AAC (N)	AAC (N)
	12669	4224	GCT (A)	TCT (S)	TCT (S)	GCT (A)	GCT (A)	GCT (A)	GCT (A)
	12855	4286	TTC (F)	TTC (F)	TTC (F)	TTC (F)	TTC (F)	CTC (L)	TTC (F)
	15289	5097	TAT (Y)	TAT (Y)	TAT (Y)	TTT (F)	TTT (F)	TTT (F)	TTT (F)
	15382	5128	CCC (P)	CCC (P)	CCC (P)	CCC (P)	CTC (L)	CCC (P)	CCC (P)
	15387	5130	GTT (V)	GTT (V)	GTT (V)	GTT (V)	GTT (V)	TTT (F)	TTT (F)
	16740	5581	GCC (A)	GCC (A)	GCC (A)	TCC (S)	GCC (A)	GCC (A)	GCC (A)
	19243	6415	ACA (T)	ACA (T)	ACA (T)	ACA (T)	ATA (I)	ACA (T)	ACA (T)
	19364	6455	ATG (M)	ATT (I)	ATG (M)	ATG (M)	ATG (M)	ATG (M)	ATG (M)
	19970	6657	AAG (K)	AAG (K)	AAG (K)	AAG (K)	AAG (K)	AAT (N)	AAG (K)
Spike	81	27	AAC (N)	AAA (K)	AAA (K)	AAA (K)	AAA (K)	AAA (K)	AAA (K)
	397	133	ACA (T)	CCA (P)	ACA (T)	CCA (P)	ACA (T)	ACA (T)	CCA (P)
	523	175	GTC (V)	GTC (V)	GTC (V)	GTC (V)	TTC (F)	GTC (V)	GTC (V)
	911	304	GAT (D)	GAT (D)	GAT (D)	GAT (D)	GCT (A)	GAT (D)	GAT (D)
	974	325	GAC (D)	GAC (D)	GAC (D)	GCC (A)	GCC (A)	GAC (D)	GAC (D)
	980	327	TCT (S)	TCT (S)	TTT (F)	TCT (S)	TCT (S)	TCT (S)	TCT (S)
	1006	336	GTA (V)	GTA (V)	GTA (V)	GTA (V)	GTA (V)	TTA (L)	GTA (V)
	1009	337	CTT (L)	CTT (L)	CTT (L)	CTT (L)	CTT (L)	TTT (F)	CTT (L)
	1096	366	GCT (A)	TCT (S)	TCT (S)	GCT (A)	GCT (A)	GCT (A)	GCT (A)
	1136	379	ACT (T)	ACT (T)	ACT (T)	ACT (T)	ACT (T)	ACT (T)	ATT (I)

1148	383	ACT (T)	ACT (T)	ACT (T)	ATT (I)	ACT (T)	ACT (T)	ACT (T)
1151-1152	384	GTT (V)	GTT (V)	GTT (V)	GTT (V)	AAT (N)	GTT (V)	GTT (V)
1168	390	GTT (V)	GTT (V)	GTT (V)	GTT (V)	GTT (V)	GTT (V)	ATT (I)
1198	400	ATC (I)	TTC (F)	ATC (I)	ATC (I)	ATC (I)	ATC (I)	ATC (I)
1306	436	TCT (S)	TCT (S)	GCT (A)	TCT (S)	TCT (S)	TCT (S)	TCT (S)
1582	528	AAC (N)	AAC (N)	AAC (N)	AAC (N)	GAC (D)	AAC (N)	AAC (N)
1835	612	GGT (G)	GGT (G)	GGT (G)	GGT (G)	GGT (G)	GTT (V)	GGT (G)
1984	662	ATC (I)	ATC (I)	ATC (I)	GTC (V)	GTC (V)	GTC (V)	GTC (V)
2132	711	GAT (D)	GAT (D)	GAT (D)	GGT (G)	GAT (D)	GAT (D)	GAT (D)
2318	773	AAG (K)	AAG (K)	AAG (K)	AAG (K)	AAG (K)	ATG (M)	AAG (K)
2323	775	GCA (A)	GCA (A)	GCA (A)	GCA (A)	CCA (P)	GCA (A)	GCA (A)
2336	779	ACT (T)	ACT (T)	ACT (T)	AAT (N)	ACT (T)	ACT (T)	ACT (T)
2482	828	GCA (A)	GCA (A)	GCA (A)	GCA (A)	GCA (A)	TCA (S)	GCA (A)
2617	873	AAT (N)	AAT (N)	AAT (N)	AAT (N)	AAT (N)	AAT (N)	GAT (D)
2659	887	AGT (S)	CGT (R)	CGT (R)	AGT (S)	AGT (S)	AGT (S)	AGT (S)
2675	892	AAA (K)	AAA (K)	AGA (R)	AGA (R)	AGA (R)	AGA (R)	AGA (R)
2678	893	AAA (K)	AAA (K)	AAA (K)	ACA (T)	AAA (K)	ACA (T)	AAA (K)
2681	894	AGG (R)	AGG (R)	AGG (R)	AGG (R)	ATG (M)	AGG (R)	AGG (R)
2770	924	CGC (R)	CGC (R)	CGC (R)	AGC (S)	CGC (R)	CGC (R)	CGC (R)
2894	965	GGT (G)	GGT (G)	GCT (A)	GCT (A)	GCT (A)	GCT (A)	GCT (A)
2898	966	TTT (F)	TTT (F)	TTA (L)	TTT (F)	TTA (L)	TTA (L)	TTT (F)
2899	967	ACT (T)	ACT (T)	TCT (S)	ACT (T)	TCT (S)	TCT (S)	ACT (T)
2905	969	GCA (A)	GCA (A)	GCA (A)	GCA (A)	GCA (A)	TCA (S)	TCA (S)
2908	970	GCG (A)	GCG (A)	GCG (A)	GCG (A)	GCG (A)	TCG (S)	TCG (S)
2915	972	TTG (L)	TTG (L)	TTG (L)	TCG (S)	TCG (S)	TTG (L)	TTG (L)
3028	1010	ATA (I)	TTA (L)	ATA (I)	ATA (I)	ATA (I)	ATA (I)	ATA (I)
3076	1026	AAG (K)	AAG (K)	AAG (K)	CAG (Q)	AAG (K)	AAG (K)	AAG (K)
3098	1033	CAT (H)	CAT (H)	CAT (H)	CAT (H)	CAT (H)	CAT (H)	CTT (L)
3500	1167	ATT (I)	ATT (I)	ATT (I)	AGT (S)	AGT (S)	AGT (S)	AGT (S)
3533	1178	ATT (I)	ATT (I)	ATT (I)	ACT (T)	ATT (I)	ATT (I)	ATT (I)
3596	1199	GCG (A)	GCG (A)	GCG (A)	GCG (A)	GCG (A)	GCG (A)	GTG (V)

	3667	1223	ATT (I)	ATT (I)	GTT (V)	GTT (V)	GTT (V)	GTT (V)	GTT (V)
	3679	1227	GTG (V)	GTG (V)	GTG (V)	TTG (L)	GTG (V)	GTG (V)	GTG (V)
	3683	1228	GTC (V)	GTC (V)	GTC (V)	GTC (V)	GTC (V)	GCC (A)	GTC (V)
	3757	1253	GAT (D)	TAT (Y)	GAT (D)	GAT (D)	GAT (D)	GAT (D)	GAT (D)
	3818	1273	AAT (N)	AAT (N)	ACT (T)	ACT (T)	ACT (T)	ACT (T)	ACT (T)
	4061	1354	TGT (C)	TTT (F)	TGT (C)	TGT (C)	TGT (C)	TGT (C)	TGT (C)
	4070	1357	TGC (C)	TGC (C)	TGC (C)	TGC (C)	TGC (C)	TTC (F)	TGC (C)
	4139-4140	1380	TTT (F)	TGA (*)	TGA (*)	TGA (*)	TGA (*)	TGA (*)	TGA (*)
	4141-4143	1381	GAA (E)	--- (-)	--- (-)	--- (-)	--- (-)	--- (-)	--- (-)
	4144-4146	1382	AAG (K)	--- (-)	--- (-)	--- (-)	--- (-)	--- (-)	--- (-)
	4147-4149	1383	GTC (V)	--- (-)	--- (-)	--- (-)	--- (-)	--- (-)	--- (-)
	4150-4152	1384	CAC (H)	--- (-)	--- (-)	--- (-)	--- (-)	--- (-)	--- (-)
	4153-4155	1385	GTG (V)	--- (-)	--- (-)	--- (-)	--- (-)	--- (-)	--- (-)
	4156-4158	1386	CAG (Q)	--- (-)	--- (-)	--- (-)	--- (-)	--- (-)	--- (-)
	4159-4161	1387	TGA (*)	--- (-)	--- (-)	--- (-)	--- (-)	--- (-)	--- (-)
ORF3	506	169	TTG (L)	TCG (S)	TTG (L)	TTG (L)	TTG (L)	TTG (L)	TTG (L)
	571	191	GAT (D)	GAT (D)	GAT (D)	TAT (Y)	TAT (Y)	TAT (Y)	TAT (Y)
	602	201	CAT (H)	CGT (R)	CAT (H)	CAT (H)	CAT (H)	CAT (H)	CAT (H)
	607	203	ATT (I)	ATT (I)	GTT (V)	ATT (I)	ATT (I)	ATT (I)	ATT (I)
Envelope	119	40	ACT (T)	ACT (T)	ATT (I)	ACT (T)	ACT (T)	ACT (T)	ACT (T)
	168	56	TTG (L)	TTG (L)	TTG (L)	TTG (L)	TTC (F)	TTC (F)	TTC (F)
	191	64	ATG (M)	ACG (T)	ATG (M)	ATG (M)	ATG (M)	ATG (M)	ATG (M)
	209	70	CCT (P)	CCT (P)	CCT (P)	CTT (L)	CCT (P)	CCT (P)	CCT (P)
Membrane	70	24	ATC (I)	ATC (I)	ATC (I)	TTC (F)	TTC (F)	TTC (F)	TTC (F)
	352	118	GCG (A)	TCG (S)	TCG (S)	GCG (A)	GCG (A)	GCG (A)	GCG (A)
	656	219	AGT (S)	AGT (S)	AGT (S)	AGT (S)	AGT (S)	AAT (N)	AGT (S)
Nucleocapsid	497	166	AGA (R)	AGA (R)	AGA (R)	ATA (I)	AGA (R)	AGA (R)	AGA (R)
	551	184	CGT (R)	CGT (R)	CGT (R)	CCT (P)	CTT (L)	CGT (R)	CGT (R)
	1130	377	GGG (G)	GGG (G)	GGG (G)	GAG (E)	GGG (G)	GGG (G)	GGG (G)
	1139	380	AAA (K)	ATA (I)	AAA (K)	AAA (K)	AAA (K)	AAA (K)	AAA (K)
